# Tumor-associated macrophage-derived chemokine CCL5 facilitates the progression and immunosuppressive tumor microenvironment of clear cell renal cell carcinoma

**DOI:** 10.7150/ijbs.74647

**Published:** 2022-07-18

**Authors:** Wenhao Xu, Yuhao Wu, Wangrui Liu, Aihetaimujiang Anwaier, Xi Tian, Jiaqi Su, Haineng Huang, Gaomeng Wei, Yuanyuan Qu, Hailiang Zhang, Dingwei Ye

**Affiliations:** 1 Department of Urology, Fudan University Shanghai Cancer Center, Shanghai, 200032, P.R. China; 2 Department of Oncology, Shanghai Medical College, Fudan University, Shanghai, 200032, P.R. China; 3 Institute of Photomedicine, Shanghai Skin Disease Hospital, School of Medicine, Tongji University, Shanghai, 200443, P.R. China; 4 Affiliated Hospital of Youjiang Medical University for Nationalities, Baise, 533000, P.R. China; 5 Department of Interventional Oncology, Renji Hospital, Shanghai Jiao Tong University School of Medicine, 200127 Shanghai, P.R. China

**Keywords:** C-C Motif Chemokine Ligand 5 (CCL5), clear cell renal cell carcinoma (ccRCC), epithelial-mesenchymal transition (EMT), tumor microenvironment (TME), tumor-associated macrophages (TAMs)

## Abstract

**Background:** Tumor-associated macrophages (TAMs) dominate the malignancy of cancers by perturbing the tumor microenvironment (TME). However, the clinical implications of heterogeneous subpopulations of TAMs in clear cell renal cell carcinoma (ccRCC) remain to be elucidated.

**Methods:** We comprehensively evaluated the prognostic implications, biological behaviors, and immunogenomics features of the C-C Motif Chemokine Ligand 5 (CCL5) expression and CCL5^+^ TME *in vitro* and in 932 real-world ccRCC patients from testing and public validation cohorts. Flow cytometry was used to examine the functional patterns of CCL5^+^ TAMs with TME cell-infiltrating characterizations.

**Results:** Our results identified distinct prognostic clusters with gradual changes in clinicopathological indicators based on CCL5 expression. Knockdown of CCL5 significantly restrained cell viability, migration capabilities of ccRCC cells, and the inhibits the proliferation and chemotaxis of THP1-derived TAMs. Mechanically, down-regulation of CCL5 arrested epithelial-mesenchymal transition by modulating the PI3K/AKT pathway in ccRCC cells. In ccRCC samples with CCL5 upregulation, the proportion of CCL5^+^ TAMs and PD-L1^+^ CD68^+^ TAMs were prominently increased, showing a typical suppressive tumor immune microenvironment (TIME). Besides, intra-tumoral CCL5^+^ TAMs showed distinct pro-tumorigenic TME features characterized by exhausted CD8^+^ T cells and increased expression of immune checkpoints. Furthermore, elevated CCL5^+^ TAMs infiltration was prominently associated with a dismal prognosis for patients with ccRCC.

**Conclusion:** In conclusion, this study first revealed the predictive value of the chemokine CCL5 on the progression and TME of ccRCC. The intra-tumoral CCL5^+^ TAMs could be applied to comprehensively evaluate the prognostic patterns as well as unique TME characteristics among individuals, allowing for the identification of immunophenotypes and promotion of treatment efficiency for ccRCC.

## Introduction

Renal cell carcinoma (RCC) is the most common and most lethal malignancy of the urinary system with an increasing annual incidence rate of approximately 1.1% worldwide 1, 2. In China, the number of kidney cancer cases increases by about 76,000 annually, and an average of 27,000 people die from RCC every year 3. Clear cell RCC (ccRCC) is now the most common histological type of RCC, accounting for approximately 80% of all RCC cases 4, 5. Nearly one-third of RCC patients are already in the advanced metastatic stages when first diagnosed which may cause a poor 5-year survival rate 6. Various strategies of immunotherapy, including the single or combinatorial use of anti-PD-1, anti-PD-L1, and anti-CTLA-4 antibodies are recommended as the mainstay of treatment for advanced RCC in recent years 7. However, immunotherapy is not equally effective for all patients. Primary and/or acquired resistance occurs in most patients receiving immunotherapy, which eventually leads to therapeutic failure 8. Furthermore, conventional targeted therapies and immune checkpoint inhibitors are limited by the challenge of overcoming the highly immunosuppressive TME in ccRCC 9. Thus, it is important to investigate the mechanisms underlying drug resistance and discover new therapeutic targets.

The tumor microenvironment (TME) is related to cancer evolution and outcomes 10, 11. Tumor-induced immune suppression largely results from numerous various underlying biological processes, many of which promote the accumulation of immune-suppressive factors into the TME, which in turn makes immunoprevention strategies less effective and contributes to tumor progression 12. The main mechanism of tumor immune evasion is facilitated by immunosuppression in the TME induced by the recruitment and polarization of suppressive tumor-associated macrophages (M2-TAMs) and regulatory T cells (Tregs); further mediated by CD4^+^, CD25^+^, FoxP3^+^ cells, and other inhibitory contextures 13. TME promote tumor growth and create a suppressive environment that interferes with treatment efficacy. Therefore, achieving effective anti-cancer therapies requires overcoming the immunosuppressive functions of the TME 14.

Interestingly, the C-C chemokine ligand 5 (CCL5) could recruit macrophages and polarize them to a M2-like subtype, hence, contributing to a suppressive TME 15. The activity of CCL5 is mediated mainly by binding to CCR5, which contributes to the activation and proliferation of natural killing cells generating C-C chemokines. CCL5 also stimulates pro-angiogenic signals by regulating endothelial cell migration, neovessel formation, and vascular endothelial growth factor secretion in tumor cells 16. Furthermore, In the TIME, TAMs-secreted CCL5 could prominently facilitates the migration, invasion, and epithelial-mesenchymal transition (EMT) of prostate cancer cells via activation of β-catenin/STAT3 signaling 17, and promotes immune evasion of colorectal cancer cells through the p65/STAT3-CSN5-PD-L1 pathway 18. In addition, CCL5 has been considered as a pro-malignancy indicator in various types of cancer, suggesting that CCL5/CCR5 axis inhibitors (such as UK-427857, Pfizer) are of great potential clinical value, and are therefore have been investigated in many preclinical studies and clinical trials 19.

In this study, we examined the potential influence of chemokine CCL5 on the clinical malignant characteristics and TME of ccRCC. We hypothesized that, in clinical practice, the CCL5^high^ TAMs could be used to comprehensively evaluate the prognostic patterns as well as the distinct immune cells infiltration of the TME among patients, allowing for the identification of immunophenotypes and effective clinical treatment strategies for ccRCC.

## Materials and methods

### Sample collection and study cohorts

The transcriptional expression profiles and matched clinicopathological data of 530 ccRCC patients were obtained from The Cancer Genome Atlas (TCGA) cohort. The "ComBat" algorithm was used to correct for batch effects of non-biotech biases and to convert FPKM values to transcripts per kilobase million (TPM) values. The clinicopathological indicators in relation to CCL5 mRNA expression of ccRCC patients in the TCGA dataset are summarized in **Supplementary Table 1**. Approved by the Clinical Research Ethics Committee of Fudan University Shanghai Cancer Center (FUSCC, Shanghai, China), this study screened a total of 413 RCC patients who underwent radical or partial nephrectomy at the Department of Urology, FUSCC with available electronic pathological and medical records. Among the included patients, 21 failed to pass the pathological quality check (since morphological manifestations not ccRCC). The FUSCC cohort was eventually composed of 392 patients with ccRCC from May 2009 to January 2020. All study designs and procedures were performed in accordance with the Helsinki Declaration II. The ethics approval and participation consent for this study was approved by the clinical ethics committee of Fudan University Shanghai Cancer Center.

### Cell culture and incubation

Two human ccRCC cell lines (A498 and 786O) and the macrophage cell line RAW264.7 were purchased from the American Type Culture Collection (ATCC, Maryland, USA). The A498, 786O, and human macrophage THP1 cell lines were cultured in RPMI-1640 (Gibco, USA) media supplemented with 10% fetal bovine serum (FBS, Hycline, Life Sciences, Shanghai, China), 100 U/ml penicillin (Beyotime, China), and streptomycin (Gibco, Grand Island, NY, USA). THP1 monocytes were treated with 100 ng/ml phorbol-12-myristate-13-acetate (PMA, Sigma-Aldrich, Shanghai, China) to induce their attachment and differentiation into macrophages after 24 h of incubation. Then, the transformation of THP1-derived macrophages into the M2 phenotype (TAMs) was induced using 10 ng/ml IL-4 (Sigma-Aldrich) for 72 h. All cells were maintained in a humidified atmosphere incubator with 5% CO_2_ at 37ºC.

### Cell transfection

Both the A498 and 786O cells have been transfected with double-stranded small-interfering RNA (siRNA) in a six-well plate using Lipofectamine 2000 reagent (RiboBio) following the manufacturer's protocol. The siRNAs against CCL5 (si-CCL5) and control were obtained from Sangon Biotech. The transfection dose used for each well was 10μl of CCL5-siRNA1 (5′- CCT CGC TGT CAT CCT CAT T -3′), siRNA-2 (5′- GAG AAG AAG TGG GTT CAA GAA -3′) or negative control RNAi (NC-siRNA, 5′-AAU UCU CCG AAC GUG UCA CGU -3′) mixed with RPMI-1640 media. The A498 and 786O cells were harvested after at least 24 h of transfection and incubation for downstream experimental analysis.

### Total mRNA extraction and Real-Time Quantitative PCR (RT-qPCR) assay

Total RNA was isolated from paired ccRCC and adjacent normal tissues and cells using a Trizol kit (Invitrogen, Carlsbad, CA). The extracted RNA was reverse-transcribed to cDNA using the PrimeScript RT reagent kit (TakaraBio, Shiga, Japan). RT-qPCR was performed using the SYBR^®^ Premix Ex TaqTM (TaKaRa) in triplicates according to the attached protocols. The comparative cycle threshold (Ct) method was used to calculate the relative expression levels of genes among groups using the 2^-ΔΔCt^ method. The primers used in the experiment were as follows: CCL5, forward 5′- AGA GCT GCG TTG CAC TTG TT -3′ and reverse 5′- GCA GTT TAC CAA TCG TTT TGG GG -3′; GAPDH, forward 5′- TCT GAC TTC AAC AGC GAC AC -3′ and reverse 5′- CAA ATT CGT TGT CAT ACC AG -3′. Relative expression was defined as the ratio of CCL5 expression in tumor/normal tissues (T/N), as previously described^20^.

### Protein isolation and Western blot analysis

Total proteins was isolated from ccRCC tumor and normal kidney samples or cancer cells using RIPA lysis buffer (Beyotime Biotechnology Shanghai, China), and was purified using the bicinchoninic acid protein assay kit (Beyotime Biotechnology, Shanghai, China). Western blot was conducted as previously described 21. The PVDF membranes were blocked and then incubated with primary antibodies for the following: CCL5 (1:1000. No.36467, Cell Signaling Technology, CST, Boston, MA, USA), E-cadherin, (1:1000, No.3195, CST), N-Cadherin, (1:1000, No.13116, CST), Snail (1:1000, No.3879, CST), Phospho-Akt, 1:1000, No. 4060, CST), Akt (1:1000, No.4691, CST), PI3K (1:1000, No.3011, CST), Phospho-PI3K (1:1000, No.4228, CST), and GAPDH primary antibody (1:1000, No.5174, CST), and a goat anti-rabbit IgG conjugated with HRP (1:3000, ab205718, Abcam) was used as the secondary antibody. Finally, the bands were visualized using the ECL-plus™ western blotting chemiluminescence detection kits (BD Biosciences, New Jersey, USA).

### Cell viability assay

The effects of CCL5 on the proliferation of ccRCC cells were determined using Cell Counting Kit-8 (CCK-8) assay. In brief, A498 and 786O cells with siRNA or negative control were seeded onto 96-well plates at a density of 2,000 cells per well. The cells were treated with CCK8 solution (KeyGEN BioTECH, Nanjing, China) for 2 h. Then the absorbance readings at 450 nm of each well were measured at 1, 2, 3, 4, and 5 days after seeding using an automatic microplate reader (TEAN, Swiss). Five replicate analyses were conducted for each sample.

### Wound-healing assay

A wound-healing assay was conducted to evaluate the migration ability of human ccRCC cells. After 48 h of transfection, the A498 and 786O cells were seeded into 6-well plates at 90% confluency. The cells were incubated and allowed to form a confluent monolayer of cells. A wound was created by gently and mechanically scratching the cell monolayer using a 200 µL Eppendorf tip. The wells were washed to remove the detached cells, while cells that remained adherent were continuously grown in serum-free media in a humidified atmosphere incubator with 5% CO2 at 37ºC. After 24 h of incubation, the 6-well plates were observed under a light microscope (400×; Nikon N-E; Nikon Corporation, Tokyo, Japan), and scratch closure changes were compared among groups using ImageJ software.

### Preparation of conditioned medium (CM) and macrophages chemotaxis assay

Cells were grown in serum-free RPMI 1640 media for 24 h after TAMs induction, then the cell culture supernatants were collected as CM according to a previous study with minor modifications 22. Human A498 and 786O ccRCC cells were seeded in a 12-well plate at a density of 5,000 cells/well. After 36 h of incubation, the CM was harvested for the treatment of macrophages. In the chemotaxis assay, macrophages were seeded at the upper chamber of the 24-well trans-well inserts at a density of 2 × 105 cells/ml. The prepared CM supplemented with 10% FBS was then added to the lower chambers in succession. After incubation for 8 h at 37℃, the macrophages that migrated into the lower chamber were fixed and stained with crystal violet dye solution (0.2% crystal violet, 20% methanol). Finally, the fixed cells in the lower chamber of the trans-well were counted using a microscope in six random fields under a light microscope (400x) to determine the cell chemotaxis capacity of macrophages.

### Assessment of immune cells infiltration in the TME of ccRCC

To assess the absolute proportion of TILs in ccRCC, we used the Cibersort deconvolution algorithm and evaluated the proportion of immune cells using support vector regression^23^. In addition, to evaluate the reliability of the deconvolution method, we used the “immuneeconv” R package that provides an integrated *P*-value from the six latest algorithms, including TIMER, xCell, MCP-counter, CIBERSORT, EPIC and quanTIseq for each sample. All the above analyses were visualized using the “pheatmap” R package.

### Least absolute shrinkage and selection operator (Lasso) regression and functional enrichment analyses

The lasso regression algorithm was used for feature selection using 10-fold cross-validation through the “glmnet” R package^24^. The CCL5^+^ TAMs signature included CCL5, CCR1, CCR3, CCR5, TNF, PF4, CD68, CD163 and CD204 (MSR1) based on the protein-protein interaction network (**Supplementary Figure 1**). The expression level of TAMs was defined as the sum of the infiltration levels of M0, M1, and M2 macrophages in ccRCC as determined by the Cibersort algorithm. To identify the underlying biological differences between CCL5^+^ TAMs clusters, we employed the limma R package to screen for differentially expressed genes (DEGs) with the threshold value set at P<0.05, |logFC|≥3 25. The expression profiles of the DEGs were extracted from the high- or low-risk of CCL5^+^ TAMs. Then, functional annotation analyses were performed to determine the potential functions of the genes using the KEGG database. The results were visualized using the ClusterProfiler R package.

### Flow cytometry (FCM) assay

Fifteen fresh tumor and adjacent normal kidney samples were resected from our institute to evaluate the levels of CCL5+ TAMs infiltration, the immune stimulatory molecules of CD8+ T cells, including TNF-α, IFN-γ, PRF-1, GZMB, and CD69, as well as the signatures of exhausted features in CD8+ T cells, including PD-L1, TIM-3, TIGIT, CTLA-4, and LAG-3. The relevant information regarding the antibodies utilized in these experiments is summarized in **Supplementary Table 2**. The samples were fixed with 1% paraformaldehyde and analyzed using Cytomic FC500 flow cytometry (Beckman Coulter, Inc.). The experiment was performed in triplicates.

### Immunohistochemistry (IHC) and multiplex immunofluorescence (mIF) staining analyses

IHC staining was performed to assess the expression levels of CCL5 using primary antibodies against CCL5 (No.36467; CST) and peroxidase-conjugated goat anti-rat IgG as previously described 26. MIF staining was conducted using the Akoya OPAL Polaris 7-Color Automation IHC kit (NEL871001KT). FFPE tissue slides were first deparaffinized in a BOND RX system (Leica Biosystems), followed by sequential incubation with primary antibodies. After this, the samples were incubated with secondary antibodies and corresponding reactive Opal fluorophores. Nucleic acids were stained with DAPI. The quantities of various cell populations were expressed as the number of stained cells per square millimeter and as the percentage of positively stained cells in all nucleated cells. Opal immunofluorescence (mIF) was implemented to determine the presence of tertiary lymphoid structures (TLS), an abundance of tumor-associated lymphocytes (TILs) and other cells, and PD-L1 expression in relation to CCL5 expression on a multispectral imaging system (Vectra® Polaris™, Shanghai, China).

### Survival analysis

The primary endpoint was overall survival (OS) and the secondary endpoint was progression-free survival (PFS) of ccRCC patients. Survival curves were constructed to assess the prognostic significance using the Kaplan-Meier method and log-rank test with 95% confidence intervals (95% CI). The cut-off value was defined via the survminer R package or median threshold. To detect the independent prognostic indicators, we assessed the hazard ratio (HR) and 95% CI using univariate and multivariate Cox logistic regression analysis and visualized the results using forest plots. The receiver operating characteristic curve (ROC) was also constructed to measure the predictive ability of the signatures.

### Statistical analysis

The statistical and graphical analyses were conducted using the SPSS software (version 23.0), GraphPad Prism software (version 8.0), or R software (version 3.3.2). One-way ANOVA was performed to compare the differences among multiple groups (≥2 groups). The student's t-test was used to compare the differences between the two groups. All hypothesis tests were two-sided and P values less than 0.05 were considered as statistically significant for all tests.

## Results

### The overall depiction of differential expression levels of CCL5 in ccRCC and normal tissues in TCGA and FUSCC cohorts

**Figure 1:**
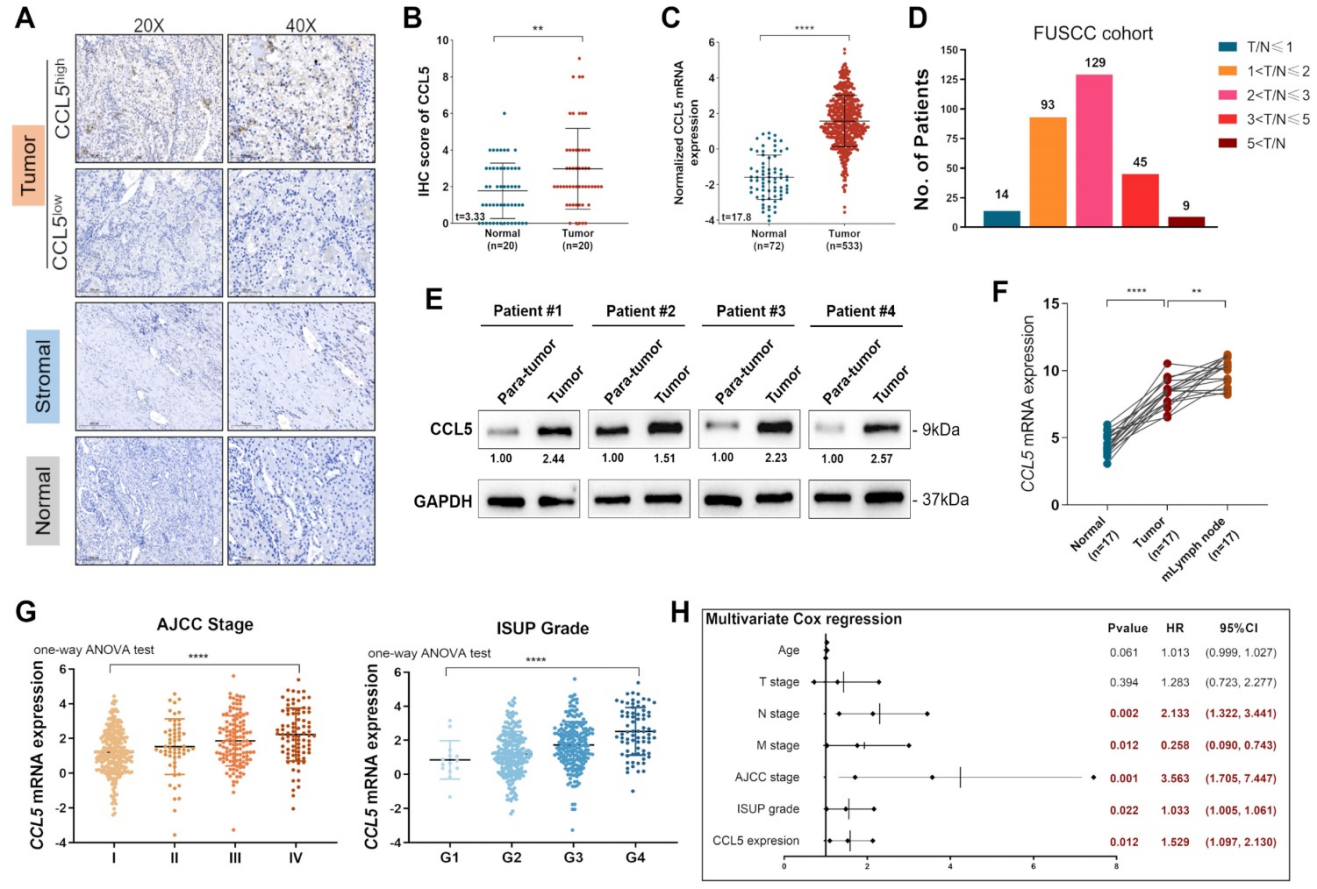
** The overall depiction of differential expression levels of CCL5 in ccRCC and normal tissues, and its correlation with clinicopathological characteristics and outcomes in TCGA and FUSCC cohorts.** (**A**) Representative immunohistochemistry images detecting CCL5 expression in paired ccRCC and normal tissues from FUSCC cohort (n=20). (**B**) Cumulative results of CCL5 IHC score from FUSCC cohort using the Students' t test. (**C**) Differential CCL5 expression in 533 tumor and 72 adjacent normal kidney samples from TCGA cohort using the Students' t test. (**D**) Distribution of paired Tumor/Normal ratio using RT-qPCR analysis from FUSCC cohort. (**E**) Western blotting assay of CCL5 expression in 4 paired fresh ccRCC and para-tumor kidney tissues. (**F**) Differential CCL5 expression in paired tumor, normal and metastatic lymph node (mlymph node) using the Students' t test (n=17). (**G**) Differential*CCL5* mRNA expression with clinical AJCC stage and pathological WHO/ISUP grade using one-way ANOV test. (**H**) Multivariate Cox regression analysis enrolling clinicopathological indicators and T/N ratio in predicting overall survival displayed in a forest plot.

To evaluate the dynamic reversible process of TME regulation by TILs and chemokines, we enrolled 20 paired ccRCC and normal tissues from the FUSCC cohort and performed IHC staining analysis to assess the protein expression of CCL5 in the tumor, stromal, and normal samples (**Figure 1A**). The results showed significant upregulation of CCL5 in ccRCC samples compared to normal tissues in the FUSCC cohort (*P*<0.01; **Figure 1B**). Similarly, we included 533 tumor and 72 adjacent normal kidney samples with available transcriptome profiles from the TCGA database and observed that *CCL5* mRNA expression was significantly higher in ccRCC compared with normal kidney samples (*P*<0.0001; **Figure 1C**). Furthermore, to validate the elevated *CCL5* mRNA expression in ccRCC samples, we inspected 290 paired ccRCC and normal tissues with clinicopathological records and follow-up data from the FUSCC cohort and designated the cluster as the discovery set. To validate the differential *CCL5* expression *in vitro*, we conducted RT-qPCR analysis using the discovery set of samples and found that the ratio of T/N was dramatically different between the distinct differential expression groups (4.8% in T/N≤1 [n=14], 32.1% in 1<T/N≤2 [n=93], 44.5% in 2<T/N≤4 [n=129], 15.5% in 4<T/N≤8 [n=45], and 3.1% in 8<T/N [n=9]; **Figure 1D**). Western blot analyses on 4 paired fresh tumor and normal samples also exhibited significantly increased CCL5 expression in 6 paired tumor tissues compared with para-tumor kidney tissues (**Figure 1E**). Interestingly, an examination of 17 paired tumor, normal, and metastatic lymph node (mlymph node) tissues showed significantly higher expression of *CCL5* in mlymph node tissues compared to the tumor and normal samples (*P*<0.01; **Figure 1F**). Taken together, we found significantly elevated transcription and protein levels of CCL5 in ccRCC samples and associated lymph node metastases compared with normal samples from both the FUSCC and TCGA cohorts.

### Differential CCL5 mRNA expression is correlated with advanced clinicopathological features in ccRCC patients from the TCGA cohort

To investigate the potential association between *CCL5* expression and different clinicopathological features, we obtained 533 ccRCC and 72 adjacent normal kidney samples from the TCGA cohort. Our results showed that higher *CCL5* expression was significantly associated with shorter survival and aggressive TNM stages (*P*<0.01; **Supplementary Figure 2, Supplementary Table 1**). Interestingly, there is a significant racial difference in *CCL5* expression (*P*=0.008). Additionally, *CCL5* mRNA expression was significantly correlated with advanced clinical AJCC stages (*P*<0.0001) showing the highest levels in advanced stage 4. Similarly, *CCL5* mRNA expression was predominantly associated with an advanced pathological WHO/ISUP grade (*P*<0.0001) showing the highest levels in progressive grade 4 (**Figure 1G**). Overall, our results show that an elevated *CCL5* mRNA expression is significantly correlated with malignant clinicopathological features and poor prognosis.

### Paired differential CCL5 expression is correlated with the clinicopathological characteristics and outcomes of patients with ccRCC from the FUSCC cohort

We measured the mRNA expression of *CCL5* in 290 patients with paired ccRCC and normal kidney samples from the FUSCC cohort. Univariate Cox regression analysis (**Supplementary Table 3**) showed that the conventional prognostic predictors, such as the lymph node and distant metastasis stage, WHO/ISUP grade, and AJCC stage, were markedly correlated to the outcomes of ccRCC patients (*P*<0.05). More importantly, an elevated *CCL5* expression T/N ratio was markedly associated with poor PFS (HR=1.642, *P=*0.002) and OS (HR=1.523, *P=*0.013). Furthermore, in our multivariate Cox regression analysis, the pN stage, pM stage, ISUP grade, and AJCC stage were still relevant to OS (pN stage: *P*=0.002; pM stage: *P*=0.012; ISUP grade: *P*<0.022; AJCC stage: *P*=0.001). Similarly, an elevated *CCL5* expression T/N ratio was remarkably associated with a shorter OS (HR=1.529, *P*=0.012) as shown by the forest plots (**Figure 1H**). Collectively, our findings showed that an elevated T/N ratio of differential *CCL5* expression was significantly correlated to malignant clinicopathological factors and poor prognosis of ccRCC patients, suggesting that the aberrant expression of *CCL5* might play an essential role in ccRCC malignancy.

### Prognostic implications of CCL5 mRNA expression in 33 TCGA cancer types

Cox regression analysis was used to understand the prognostic value of CCL5 mRNA expression in 33 different TCGA cancer types. The results were visualized using forest plots. Significantly, CCL5 expression was able to distinguish poor prognosis and progressiveness in patients with kidney renal clear cell carcinoma (KIRC), brain lower-grade glioma (LGG), sarcoma (SARC), skin cutaneous melanoma (SKCM), uterine corpus endometrial carcinoma (UCEC), and uveal melanoma (UVM) among others (*P*<0.05; **Supplementary Figure 3A-B**). In addition, histograms showed the predictive value of CCL5 expression in predicting advanced clinical stages and aggressive pathological grades (**Supplementary Figure 3C-D**). Overall, the results revealed a prominent role of CCL5 expression in association with clinical and pathological indicators, especially for ccRCC patients.

### Large-scale samples identify prognostic implications of CCL5 expression patterns and sensitivity of targeted drug in patients with ccRCC

**Figure 2:**
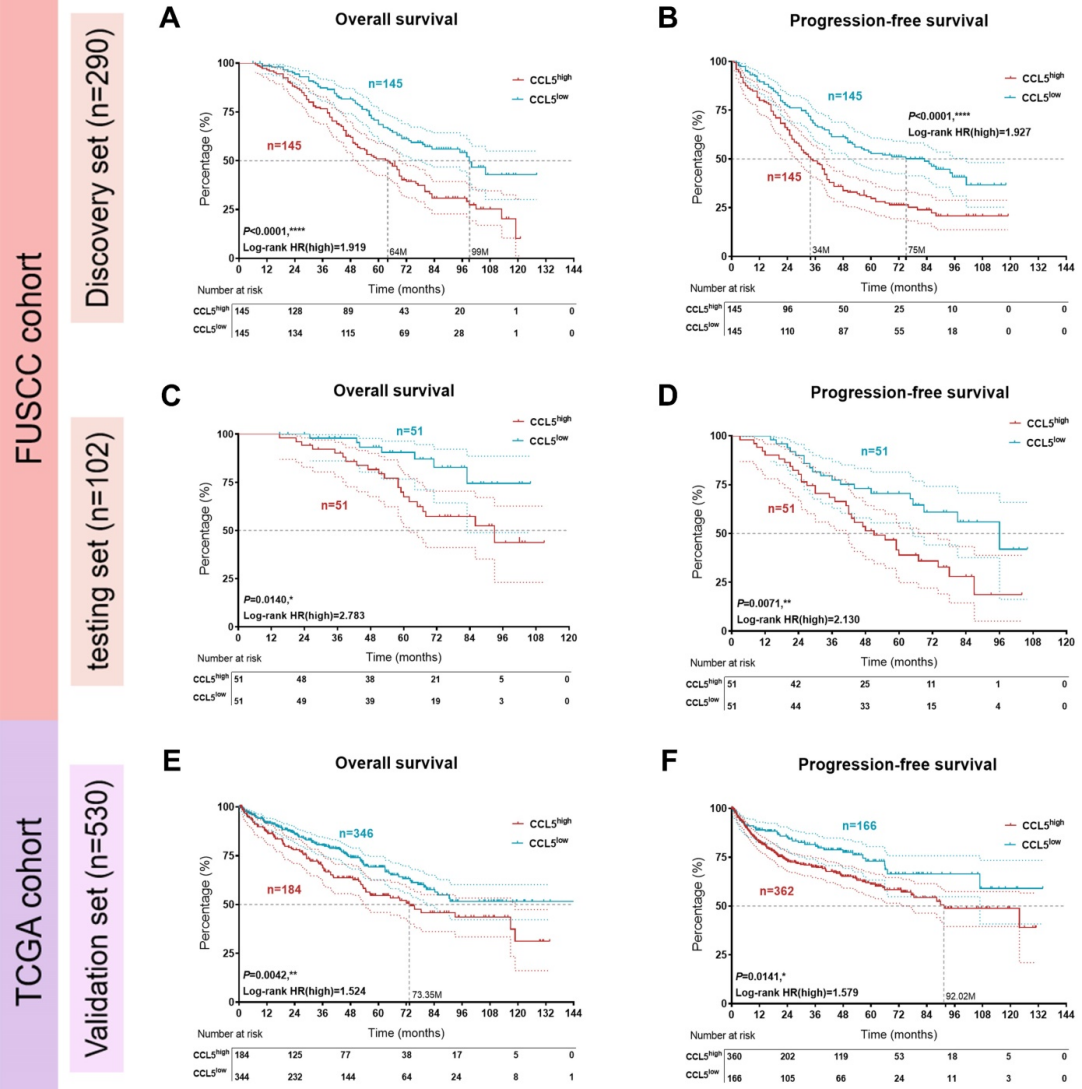
** Prognostic implications of CCL5 expression patterns in large-scale patients with ccRCC from the FUSCC and TCGA cohorts.** (**A-B**) Kaplan-Meier and log-rank tests identify predictive value of CCL5 expression in prognostic prognosis in discovery set (n=290) from FUSCC cohort. (**C-D**) Kaplan-Meier and log-rank tests identify prognostic value of CCL5 expression in testing set (n=102) from FUSCC cohort. (**E-F**) Kaplan-Meier and log-rank tests identify prognostic value of CCL5 expression in validation set (n=530) from TCGA cohort.

Three datasets with available clinical follow-up and clinicopathological data (discovery set, n=290, from FUSCC; testing set, n=102, from FUSCC; and validation set, n=530, from TCGA) were included in survival analyses. The comprehensive prognostic implications of *CCL5* expression for 290 Asian ccRCC patients from the FUSCC cohort are shown in** Figure 2A-B**. The results showed that higher *CCL5* expression significantly predicts poor survival and enhanced cancer progression (OS: *P*<0.001, HR=1.919; PFS: *P*<0.001, HR=1.927). Similarly, in the testing set from the FUSCC cohort, CCL5^high^ was also markedly tied with poor OS and progressive PFS for 102 ccRCC patients (OS: *P*=0.014, HR=2.783; PFS: *P*=0.0071, HR=2.130; **Figure 2C-D**). Additionally, for 530 samples from the validation TCGA cohort, elevated *CCL5* expression was highly linked to poor outcomes and advanced progression (OS: *P*=0.0042, HR=1.524; PFS: *P*=0.0141, HR=1.579; **Figure 2E-F**). These findings revealed that *CCL5* expression allowed the prognostic stratification of patients in both the FUSCC and TCGA cohorts.

The semi-inhibitory concentration (IC50) is a vital indicator for evaluating drug efficacy and treatment responses for individuals based on transcriptome profiles from the Genomics of Drug Sensitivity in Cancer (GDSC) database. The distribution of IC50 scores for conventional tyrosine kinase inhibitors was evaluated in ccRCC samples with differential CCL5 expression levels. The findings suggested that patients with high CCL5 expression could exhibit significantly favorable clinical responses to Axitinib (*P*=0.013) and Sunitinib (*P*=1.7e-06), with poor responses to Sorafenib (*P*=0.00055; **Supplementary Figure 4**). Based on these findings, we hypothesized that the potential malignant effects induced by CCL5 might break the balance between the anti- and pro-tumorigenic effects as regulated by the aberrant immune cells infiltration and vascular proliferation signals.

### Inhibition of CCL5 restrains proliferation and migration capacities of ccRCC cells

**Figure 3:**
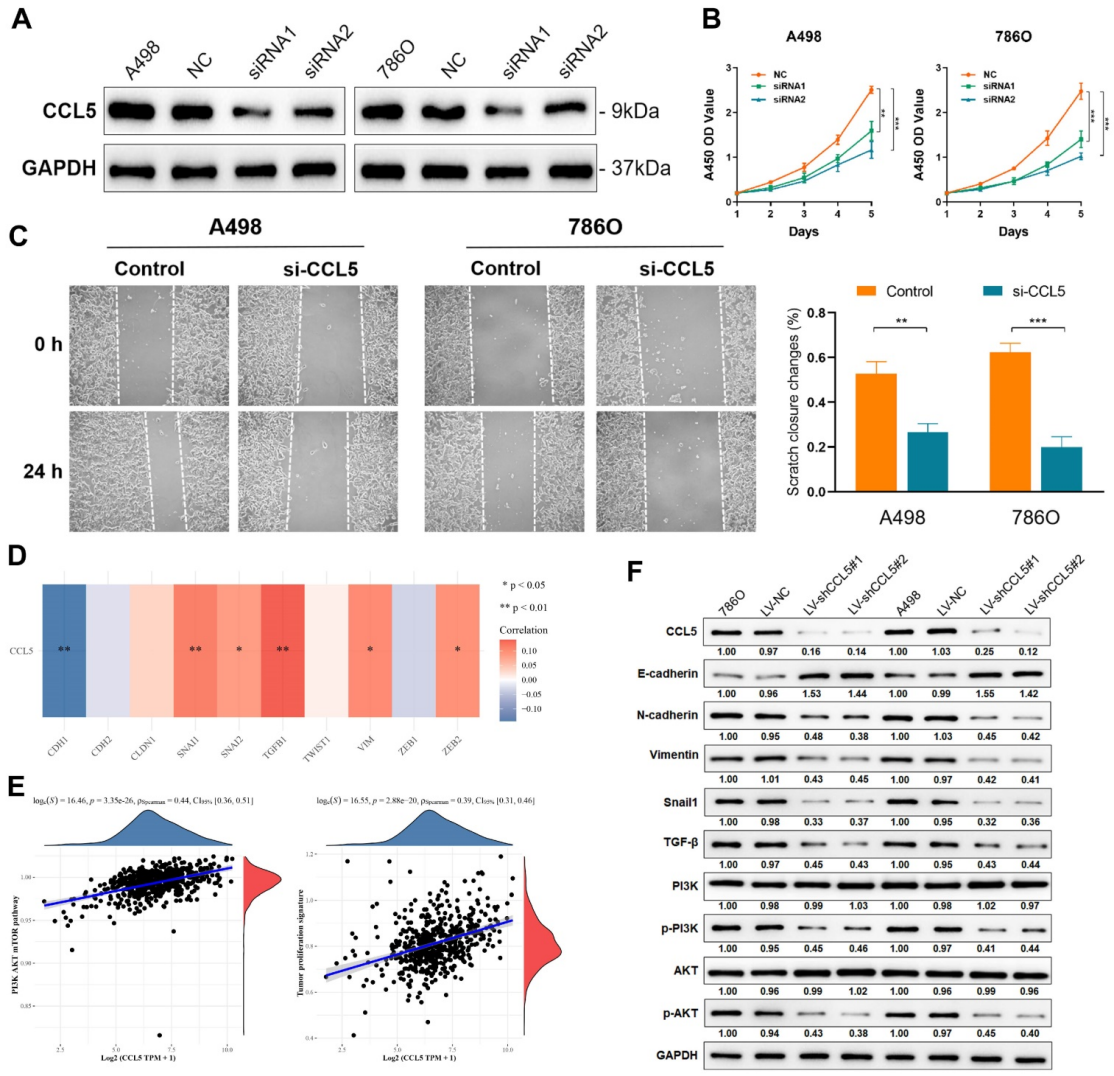
** Inhibition of CCL5 restrains proliferation, migration capacities of ccRCC cells and chemotaxis of macrophages.** (**A**) Western blotting assay of CCL5 expression in transfected A498 and 786O cell lines. The data represent three independent experiments. (**B**) Cell viability of negative control and transfected ccRCC cells using CCK-8 assay. (**C**) Effect of CCL5-siRNA transfection on cell migration was determined using wound-healing assay. Histograms and Students's t test were used to evaluate the significance between two groups. (**D**) Correlation of CCL5 and epithelial-mesenchymal transition (EMT)-related markers in 533 patients with ccRCC from TCGA were detected by Spearson's correlation analysis. (**E**) The linear relationship of CCL5 expression with PI3K/AKT signaling pathway and tumor proliferation signature by Spearson's correlation. (**F**) Using Western blotting assay, expression of crucial markers in the EMT and PI3K/AKT signaling pathways were assessed.

To validate the knockdown of CCL5, we measured its expression levels in A498 and 786O cells transfected with normal control, siRNA1, and siRNA2. A significant decrease in CCL5 expression was found in CCL5-RNAi-transfected A498 and 786O cells (*P*<0.05; **Figure 3A**). Using these cell lines, we performed CCK8 and wound healing assays to evaluate cell proliferation and migration, respectively. The results showed a significantly suppressed cell viability in the siRNA1 and siRNA2-transfected groups compared with the normal control group (*P*<0.05; **Figure 3B**). Furthermore, the wound healing assay results showed that after 24 h, the changes in the scratch closure were significantly higher in the si-CCL5 group compared to the normal control group (**Figure 3C**). These results revealed that knocking down CCL5 significantly restrains cell viability and migration capacities *in vitro* and showed that CCL5 expression could distinguish aggressiveness in ccRCC.

### Downregulation of CCL5 inhibits the EMT process via the PI3K-AKT pathway in ccRCC cells

To elucidate the underlying mechanism behind the effects of CCL5 in the proliferation and migration capacities of ccRCC cells, we first determined the correlation between CCL5 and the EMT-related markers in 533 ccRCC patients from the TCGA dataset. CCL5 expression showed a significantly negative association with the typical epithelial cell marker, E-cadherin (CDH1; *P*<0.01), and a remarkably positive relationship with mesenchymal markers that reflect increased cell motility and ability to invade and metastasize, including Snail1, Snail2, TGF-β1, VIM, and ZEB2 (*P*<0.05; **Figure 3D**). Next, we investigated the upstream signaling pathway and downstream biological behaviors which are related to these relationships. We found that CCL5 expression has a significantly strong positive linear relationship with the PI3K/AKT signaling pathway and a tumor proliferation signature (Spearson's correlation value = 0.44 and 0.39, respectively, *P*<0.0001;** Figure 3E**). Using Western blot, we found a significantly increased expression of E-cadherin and decreased expression of mesenchymal markers, such as N-cadherin, Vimentin, and Snail, in CCL5-knockdown ccRCC cells (**Figure 3F**). In addition, we observed that the phosphorylation levels of PI3K and AKT were remarkedly suppressed in the CCL5-knockdown cells, with an unaltered expression of PI3K and AKT between groups. Based on these findings, we speculated that CCL5 could regulate EMT by activating the PI3K/AKT pathway in ccRCC cells.

### Inhibition of CCL5 restrains the proliferation and chemotaxis of THP1-derived TAMs

**Figure 4:**
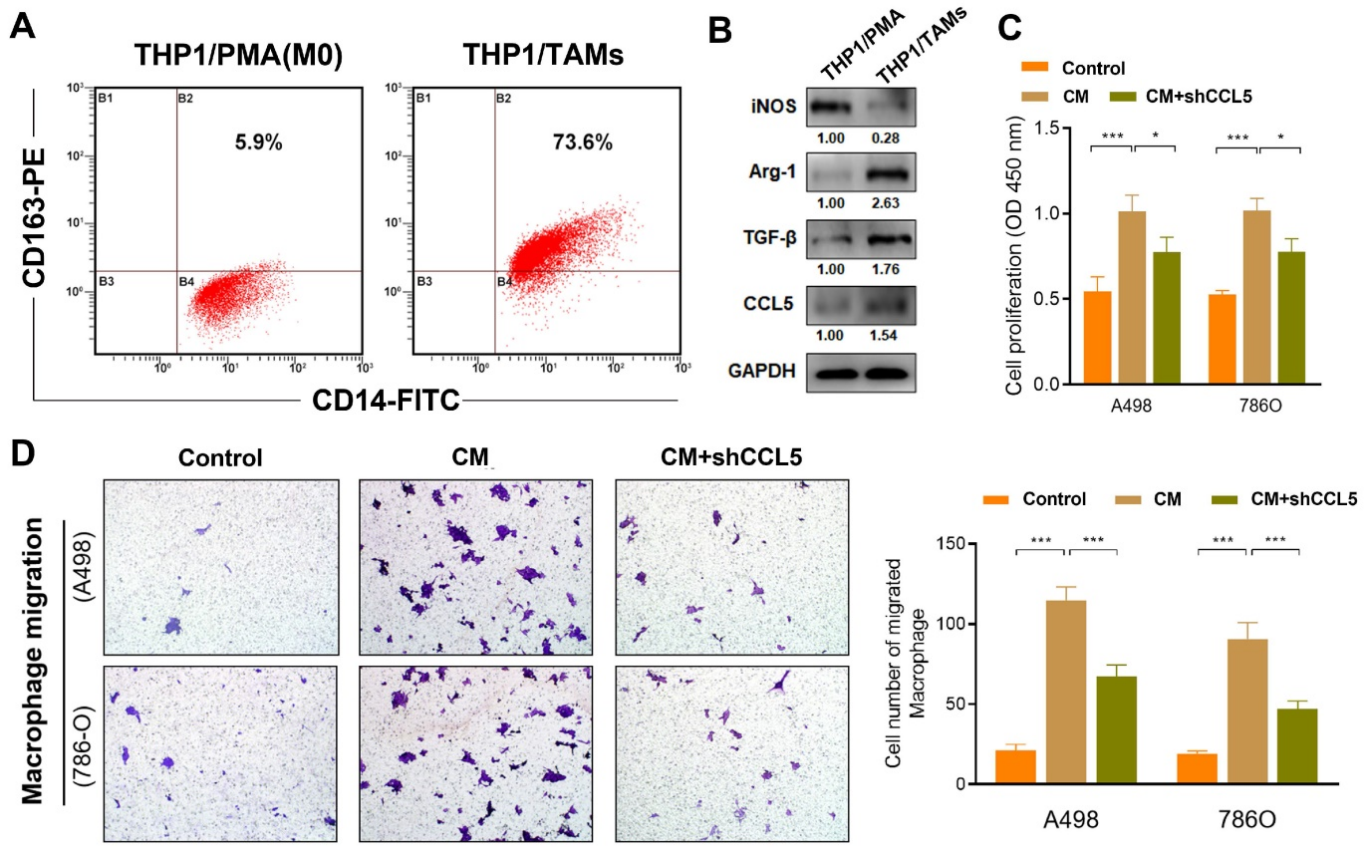
** Inhibition of CCL5 restrains proliferation and chemotaxis of THP1-derived TAMs.** (**A**) THP1 cells were induced differentiation into macrophages (M0) using PMA treatment and further transformed into M2 phenotype macrophages (specifically THP1-derived TAMs) induced by IL-4. (**B**) Using Western blotting assay, expression of iNOS, Arg-1, TGF-β, and CCL5 were assessed in PMA induced THP1 cells and THP1-derived TAMs. (**C**) CCK8 assay was supplemented to explore the effect of CCL5 on the proliferation ability of THP1-derived TAMs. (**D**) Representative macrophage migration images of the control group, conditioned medium (CM) of tumor cells group and the CM of CCL5-knockdown ccRCC cells. Histograms and Students's t test were used to evaluate the significance between two groups.

In this study, the differentiation of THP1 cells into macrophages (M0) was induced using PMA treatment. Further transformation into M2 phenotype macrophages (specifically THP1-derived TAMs) was facilitated by IL-4 treatment (**Figure 4A**). The THP1-derived TAMs showed an elevated expression of the M2 phenotype markers, Arg-1 and TGF-β, as well as the decreased expression marker iNOS (traditional marker of M1 macrophages), which reflected successful induction of THP1-derived TAMs. Interestingly, western blot also revealed that CCL5 expression is higher in THP1-derived TAMs compared with THP1 cells treated with PMA (**Figure 4B**). Next, to explore the effect of CCL5 on the proliferation ability of THP1-derived TAMs, a CCK8 assay was performed. The results showed that the CM from the THP1-derived TAMs significantly promoted cell viability (*P*<0.001; ​**Figure 4C**). Notably, we found a significantly decreased proliferation of THP1-derived TAMs in the CCL5-knockdown group compared to the CM-treated THP1-derived TAMs group (*P*<0.05). Furthermore, to determine the effect of CCL5 on macrophage chemotaxis, murine peritoneal macrophages were seeded into the upper trans-well chambers and treated with CM from A498 and 786O cells for 12 h. The results showed that the migration of macrophages treated with either A498-vector-CM or 786O-vector-CM was significantly increased compared with treatment from the negative control group (**Figure 4D**). The CM from A498-vector-CM and 786O-vector-CM strikingly increased the degree of THP1-derived TAMs chemotaxis compared with the CM from the A498-vector and 786O-vector cells. Altogether, these results validated that the inhibition of CCL5 expression could restrain the proliferation and migration of THP1-derived TAMs *in vitro*.

### Enrichment of CCL5 in ccRCC microenvironment and its distribution on TAMs

**Figure 5:**
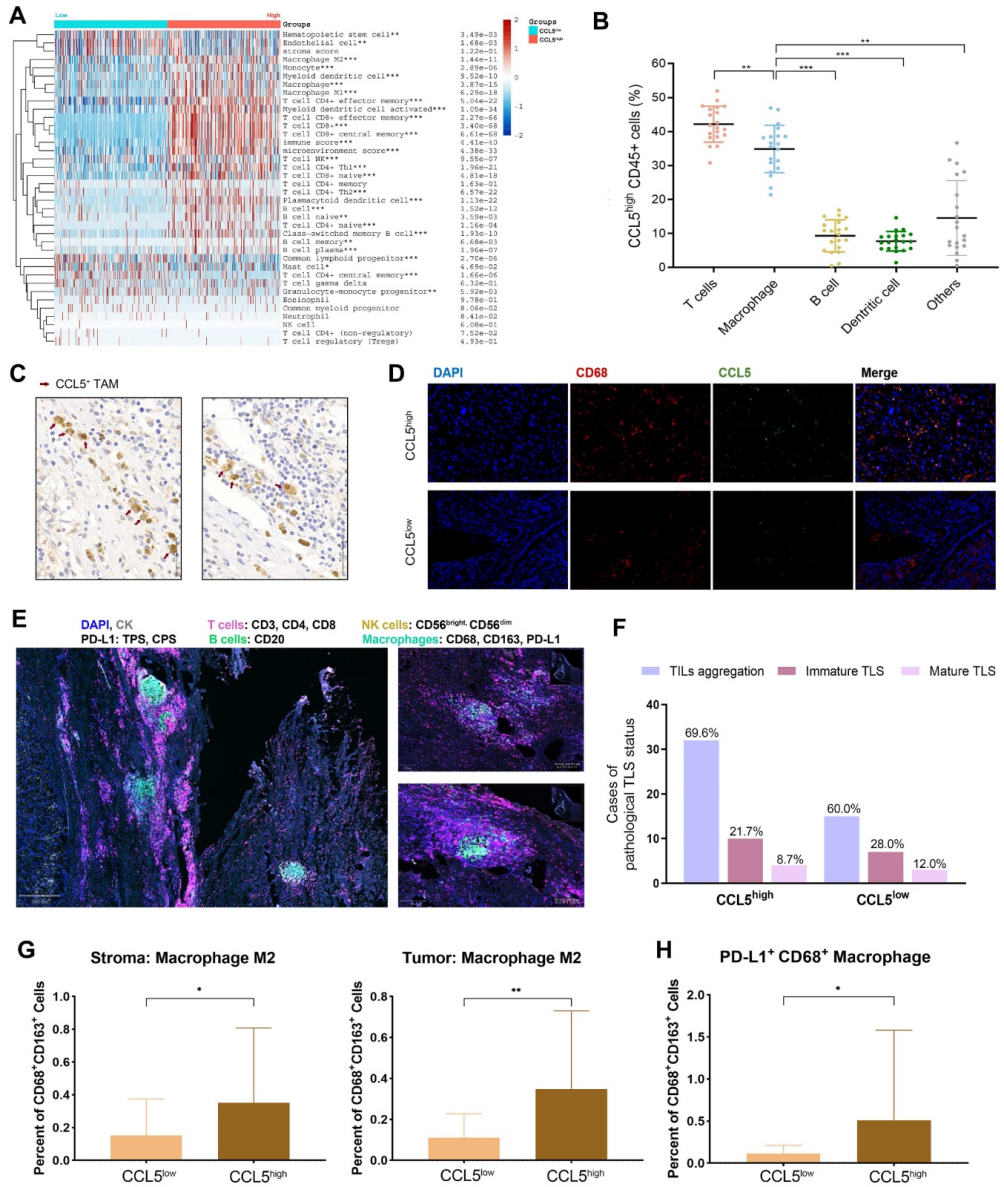
** Perturbation of CCL5 in immune microenvironment of ccRCC and its distribution on TAMs.** (**A**) Differential *CCL5* expression distinguished the individual with ccRCC into two immunophenotypes using unsupervised clustering xCell algorithm for patients with ccRCC from TCGA database. (**B**) Cumulative results showing subpopulations gated on CCL5^high^ CD45^+^ leucocytes from fresh human ccRCC samples (n=20). (**C**) Representative immunohistochemistry images of CCL5 expressed macrophages. (**D**) Co-labeled with macrophage marker CD68 (red) and CCL5 (green) in ccRCC tissues. Nuclei were counterstained blue with DAPI. The right panel was merged by the aforementioned three images. (**E**) Opal multiplex immunofluorescence (mIF) was implemented to determine presence of tertiary lymphoid structures (TLS), abundance of tumor associated lymphocytes (TILs) and other cells, and PD-L1 expression in accordance to CCL5 expression on a multispectral imaging system in ccRCC tissues. (**F**) Histogram showing the aggregation of TILs and presence of TLS in ccRCC samples with different CCL5 expression levels. The bar graphs means the number of cases with TILs aggregation, immature TLS or mature TLS. The percentages written on top of each group means the proportion of this group of samples in the CCL5high or CCL5high group. (**G**) Histogram showing the percent of CD68^+^CD163^+^ macrophage M2 in stromal and tumor samples with different CCL5 expression levels using unpaired t test. (**H**) Histogram showing the percent of PD-L1^+^ macrophages in ccRCC samples with different CCL5 expression levels using unpaired t test.

The above findings demonstrated that CCL5 plays a crucial regulatory role in re-shaping the TME characteristics. Considering the individual intra-tumoral heterogeneity of ccRCC, we established a scoring system for the precise quantification of the immunogenomic characterizations using lymphocyte-related genes through the CIBERSORT algorithm (**Supplementary Table 4**). The differential expression of *CCL5* significantly distinguished individual ccRCC patients into two immune-infiltrated clusters (“hot” and “cold”) as determined by the xCell algorithm for ccRCC patients from the TCGA database (**Figure 5A**). Notably, a significantly increased abundance of macrophages, CD4^+^ T cells, CD8^+^ T cells, and B cells were found in the CCL5^high^ group (*P*<0.01). To determine the specific distribution of *CCL5* in the immune microenvironment of ccRCC, the co-localization of CCL5 with the classic signatures of TILs was assessed. Results of flow cytometry demonstrated that CCL5 was largely expressed by T cells and macrophages compared with B cells, dendritic cells, and other TILs (**Figure 5B**). We next examined the distribution of CCL5 expression in the ccRCC patients from the training cohort using IHC staining. Consistent with previous findings, we observed that CCL5 was mainly enriched on the surface of TAMs (**Figure 5C**). In addition, immunofluorescence analysis of human ccRCC tissues revealed that CCL5 was co-localized with CD68, a macrophage marker (**Figure 5D**).

### CCL5 expression correlates with an elevated abundance of TILs and M2 macrophages in ccRCC samples

Multiple tumor cell-derived signaling molecules have crucial roles in remodeling the TME, inducing key features of TAMs polarization, and promoting tumor growth. To determine the phenotypic role of CCL5 in the TME landscape of ccRCC, we performed a precise batch quantitative analysis of various immune cells in the TME of FFPE ccRCC samples using the Opal^TM^ multi-label staining experiments and Akoya multispectral imaging to obtain images with high signal-to-noise ratios. Representative images of tertiary lymphoid structures (TLS) visualized using multi-label IHC analysis are shown in **Figure 5E**. It was found that samples with high CCL5 expression exhibited higher aggregation of TILs, but a reduced proportion of immature and mature TLS compared to CCL5^low^ samples (**Figure 5F**). In addition, there was a high level of infiltration of M2 macrophages in the stromal and tumor tissues with high expression of CCL5. Furthermore, the proportion of PD-L1^+^ CD68^+^ TAMs was also prominently increased in CCL5^high^ samples (**Figure 5G-H**), showing a typical suppressive TME.

### Intra-tumoral CCL5^+^ TAMs expose a distinct subset with pro-tumorigenic exhausted features and impaired total CD8^+^ T cell function in ccRCC

**Figure 6:**
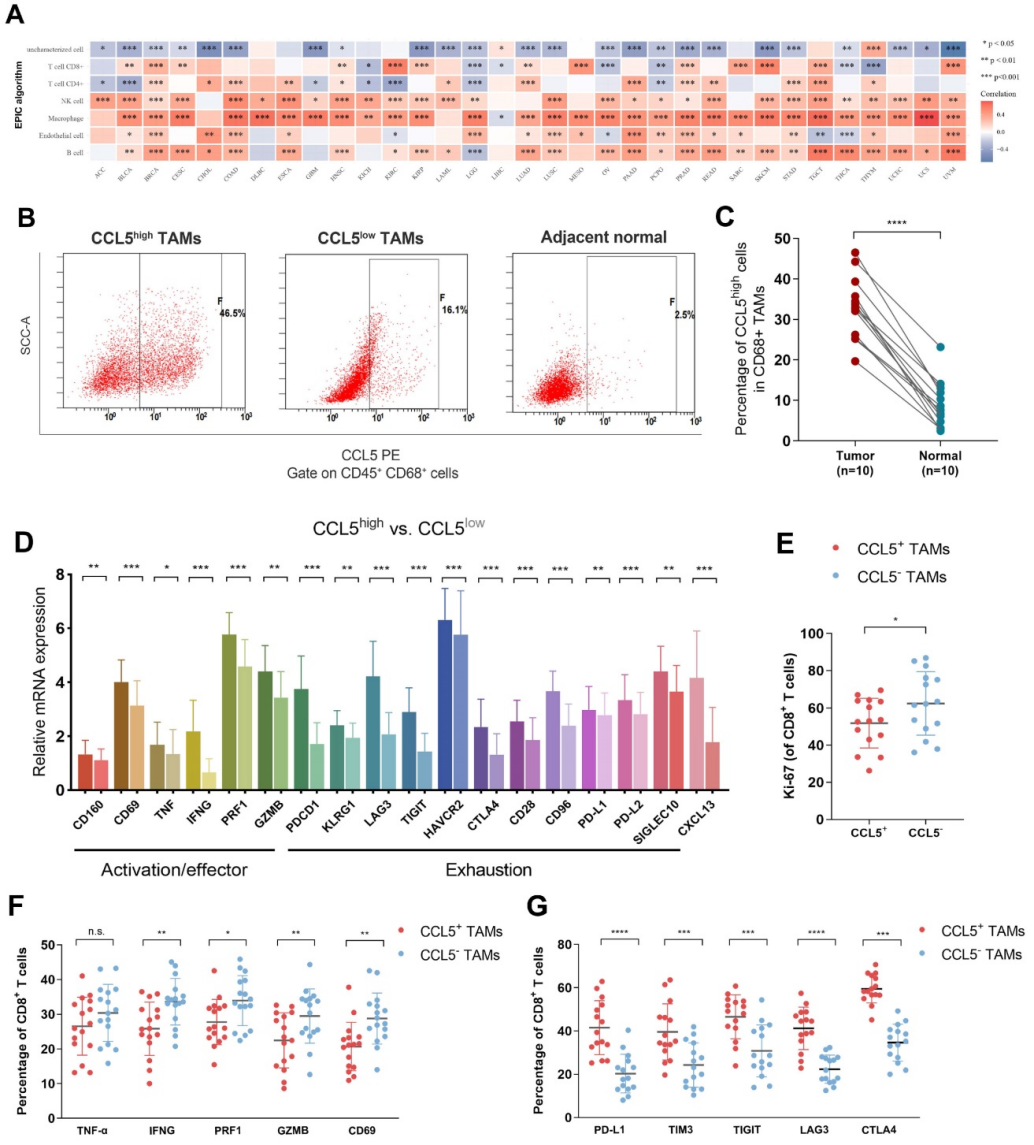
** Intra-tumoral CCL5+ TAMs exposed a distinct subset with pro-tumorigenic exhausted features and impaired total CD8^+^ T cell function of ccRCC** (**A**) We next evaluated the Relationship between transcriptomic *CCL5* expression and cells infiltration level in the TME among 33 cancers in the TCGA database using Spearson's correlation analysis. (**B-C**) Representative images and histogram of flow cytometry showed infiltration of CCL5^high^ CD68^+^ cells in total CD45^+^ CD68^+^ cells in tumor and peritumor tissues (n=15). Data were analyzed using the Students' t test. (**D**) Differential level of immune checkpoints activation, exhaustion signatures and CXCL13 expression were compared based on Cibersort algorithm between CCL5^high^ and CCL5^low^ ccRCC samples from TCGA cohort using unpaired t test. (**E**) Differential proliferation biomarker Ki-67 expression in CD8^+^ T cells within CCL5^+^ or CCL5^-^ TAMs ccRCC fresh tumor tissues using flow cytometry analysis and unpaired t test. (**F**) Differential immunity stimulatory effectors in CD8^+^ T cells within CCL5^+^ or CCL5^-^ TAMs ccRCC fresh tumor tissues using flow cytometry analysis and unpaired t test. (**G**) Differential immune checkpoints molecules within CCL5^+^ or CCL5^-^ TAMs ccRCC fresh tumor tissues using flow cytometry analysis and unpaired t test.

We next evaluated the relationship between *CCL5* expression and the TILs landscape in the TME of ccRCC. A significant association between transcriptomic *CCL5* expression and macrophages has been identified among 33 cancer types in the TCGA database. In the TCGA testing cohort, *CCL5* expression showed a marked correlation with increased CD8^+^ T cells, NK cells, and B cells, and decreased CD4^+^ T cells (**Figure 6A**). These findings further confirmed our hypothesis that the anti-tumor immune effect-dependent T cell killing in ccRCC might be regulated by the CCL5-overexpressing TAMs to reduce its exhaustion features, thereby promoting the immune escape activity of tumor cells. Consequently, we found that ccRCC specimens exhibited a significantly elevated abundance of CCL5^high^ TAMs compared with the adjacent normal samples (**Figure 6B-C**).

To explain the pro-tumorigenic merit of CCL5-overexpressing TAMs, we aimed to investigate the effects of intra-tumoral CCL5^+^ TAMs on the immune contexture of ccRCC. The Cibersort algorithm was used in 530 ccRCC samples from TCGA and compared the differential levels of immune checkpoints activation (CD160, CD69, TNF, IFNG, PRF1, and GZMB) and exhaustion (PDCD1, KLRG1, LAG3, TIGIT, HAVCR2, CTLA4, CD28, CD96, PD-L1, PD-L2, and SIGLEC10) signatures. The results showed the elevated expression levels of activation/effector and exhaustion signatures, and CXCL13 in CCL5^high^ compared with CCL5^low^ samples (**Figure 6D**). To validate the remarkable differential status of TIME in relation to CCL5 expression, a FCM assay was performed on 30 fresh ccRCC tumor tissues to assess the impact of intra-tumoral CCL5^+^ TAMs on TME functions. Our findings showed a significantly lower proliferation ability of CD8^+^ T cells in CCL5^+^ TAMs compared to CCL5^-^ TAMs clusters (*P*<0.05; **Figure 6E**). In addition, CCL5^+^ TAMs within ccRCC tissues exhibited lower levels of immune stimulatory effective molecules (IFNG, PRF1, GZMB, and CD69) and increased expression of immune inhibitory checkpoints (PD-L1, TIM3, TIGIT, LAG3. and CTLA4) compared with CCL5^-^ TAMs (*P*<0.05; **Figure 6F-G**). Taken together, intra-tumoral CCL5^+^ TAMs exhibited a distinct pro-tumorigenic exhausted state of CD8^+^ T cells and promoted an immunoevasive contexture in ccRCC.

### Accumulation of CCL5^+^ TAMs distinguished clinical outcomes in patients with ccRCC

**Figure 7:**
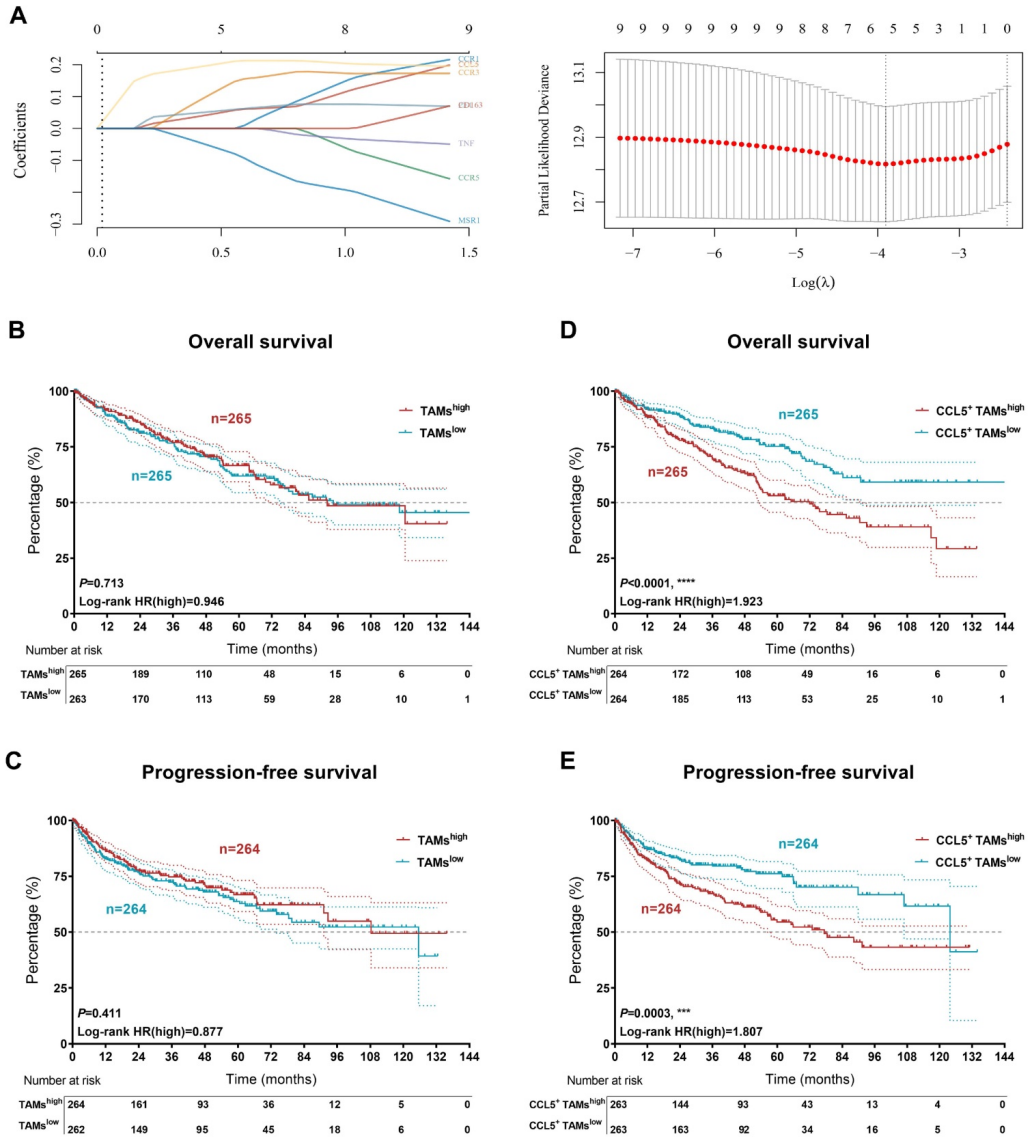
** Accumulation of CCL5^+^ TAMs distinguished clinical outcomes in patients with ccRCC.** (**A**) The necessary transcription signatures composing CCL5^+^ TAMs were enrolled in Lasso regression analysis to establish the CCL5^+^ TAMs model for 530 patients with ccRCC from validation TCGA cohort. (**B-C**) Kaplan-Meier and log-rank tests identify prognostic value of TAMs infiltration alone in validation set (n=530) from TCGA cohort. (**D-E**) Kaplan-Meier and log-rank tests identify prognostic value of CCL5^+^ TAMs infiltration in validation set (n=530) from TCGA cohort.

Kaplan-Meier curves were constructed to demonstrate the prognostic value of CCL5^+^ TAMs in the TCGA validation cohort. The expression level of TAMs was defined as the sum of the infiltration levels of M0, M1, and M2 macrophages in ccRCC as determined by the Cibersort algorithm. The necessary transcription signatures constituting the CCL5^+^ TAMs were enrolled in Lasso regression analysis to establish the CCL5^+^ TAMs model for ccRCC (**Figure 7A**). Interestingly, TAMs alone failed to determine the overall outcomes and progression of the ccRCC patients in the validation cohort (OS, *P*=0.713; PFS, *P*=0.411;** Figure 7B-C**). Although there is a high tumor heterogeneity for TAMs within ccRCC, we found that increased abundance of tumor-infiltrating CCL5^+^ TAMs represented worse OS and PFS compared to tumors with lower CCL5^+^ TAMs-infiltration in ccRCC patients (OS, *P*<0.0001, HR=1.923; PFS, *P*=0.0003, HR=1.807;** Figure 7D-E**). Additionally, the DEGs extracted based on the differential infiltration levels of CCL5^+^ TAMs in ccRCC were integrated and functionally annotated using KEGG analyses (**Supplementary Figure 5**). Interestingly, pathway analyses showed that the DEGs were significantly involved in the activation of the PD-L1/PD-1 checkpoint pathway in cancer. Conclusively, these findings revealed that the infiltration level of pro-tumorigenic CCL5^+^ TAMs cells could serve as an independent predictor for evaluating the immunoevasive TME and clinical outcomes of ccRCC.

## Discussion

Interestingly, in the Chinese population, ccRCC, the most common and malignant subtype of RCC, exhibits distinct proteomic characteristics and immune phenotypes 27. Increasing evidence suggests that the malignant behaviors and treatment resistance of cancers are heavily governed by the crosstalk between the TME and tumor cells 28. Chemokines are crucial in modulating the activity and tolerance status of tumors, as well as the abundance and cellular differentiation of TILs involved in pro- and anti-tumorigenic immune activities 29-32. Previous studies found high CCL5 expression in PBRM1^MUT^ ccRCC patients that exhibited poor clinical outcomes and increased mast cell infiltration 33. However, the characteristics and implications of CCL5 expression patterns on TAMs and immunosuppressive TMEs in ccRCC need further investigation.

In this study, we identified two distinct CCL5 expression patterns in large-scale ccRCC cohorts, which were associated with prominent differences in clinicopathological features of TME. More specifically, we found that the significantly upregulated CCL5 expression in ccRCC was associated with disease progression and suppression of immune stimulatory factors, corresponding to the immune-infiltrated phenotype. As we have previously observed, even in cases of immune-infiltrated TME with the presence of mature TLS, TILs rarely appear in the stromal components 34. Furthermore, CCL5 has been demonstrated as a predictive biomarker for evaluating the migration of tumor cells and induction of macrophage infiltration, thereby contributing to the aggressive progression of lung cancer 35. Consistently, in this study, we showed that the aggressive CCL5^high^ phenotype is prominently correlated with the progressive malignancy, migration and proliferation of tumor cells, and chemotaxis of macrophages. We suggest that our findings can contribute to the development of new drugs and targeted treatment strategies for ccRCC patients.

The immune escape and dysregulation of metabolic phenotypes play critical roles in cancer progression^36^, ^37^. For example, tumor glycolysis affects the TME, which in turn is a major obstacle to the successful targeting of cancer by anti-tumor immunotherapies and other therapies 21, 38. Studies have shown that TAMs essentially belong to the M2 polarized macrophages, with a downregulated IL-12 and an upregulated IL-10 expression on the cell surface, as well as upregulated TGF-β and CCL22 39. TAMs were shown to drive T cell responses in the TME and promote tumor progression and metabolic abnormalities by mediating immune escape through the PD-1/PD-L1 axis 40. In this study, not only cells with CCL5 expression in ccRCC samples showed a typical suppressive TIME, but intra-tumoral CCL5^+^ TAMs also exposed distinct pro-tumorigenic exhausted state of CD8^+^ T and promoted an immunoevasive contexture in ccRCC. Indeed, the abundance of TAMs in tumor tissues was directly correlated with the tumor vascularity and the robustness of tumor invasion, the metastasis status, and the formation of an immunosuppressive TME 41. In relation to this, TAMs-targeted treatment combined with conventional therapies has been shown to offer beneficial therapeutic approaches for future challenges in solid tumors 42.

Tertiary lymphoid structures (TLS) are ectopic lymphoid structures that form under chronic inflammatory conditions within tumors^43^. TLS primarily consists of B cells, T cells, dendritic cells, and a high volume of vasculature. Furthermore, TLS displays different levels of organization, ranging from locally concentrated immune cell aggregates, to well-defined B cell follicles, to mature follicles containing germinal centers 44. With the advancements in cancer research, it has been found that the infiltration of TLS is tightly correlated with improved outcomes from cancer immunotherapy in patients with solid tumors and is a potential prognostic signature. Moreover, studies have shown that for most tumors, the infiltration of TLS in the TME offers a better prognosis 45, 46. However, due to intra-tumoral heterogeneity, the degree of immune cell infiltration and the predictive value of TLS in evaluating the prognosis and treatment of tumors differs substantially 47. CCL5 was previously identified as one of the 12-chemokine signatures for predicting TLS as determined by evaluating pan-cancer transcriptomic profiles 48. Furthermore, it was proven to be a part of a hub of prognostic genes for ccRCC in our previous findings 34. We confirmed the abundance of TILs aggregation and TLS in ccRCC and demonstrated a potential oncogenic role of CCL5 expression in ccRCC. Our findings may help in the development of immunotherapies and provide novel insights into the management of long-term treatment strategies for ccRCC. Moreover, TLS can effectively activate intra-tumoral cytotoxic CD8^+^ T cells to expand clonally and infiltrate into tumors by increasing CCL5 or CXCL10 secretion. After sensitization, the TME maintains an inflamed state of T cells, allowing tumor cells to respond positively to anti-PD-1 therapy with durable therapeutic effects 49.

**Figure 8:**
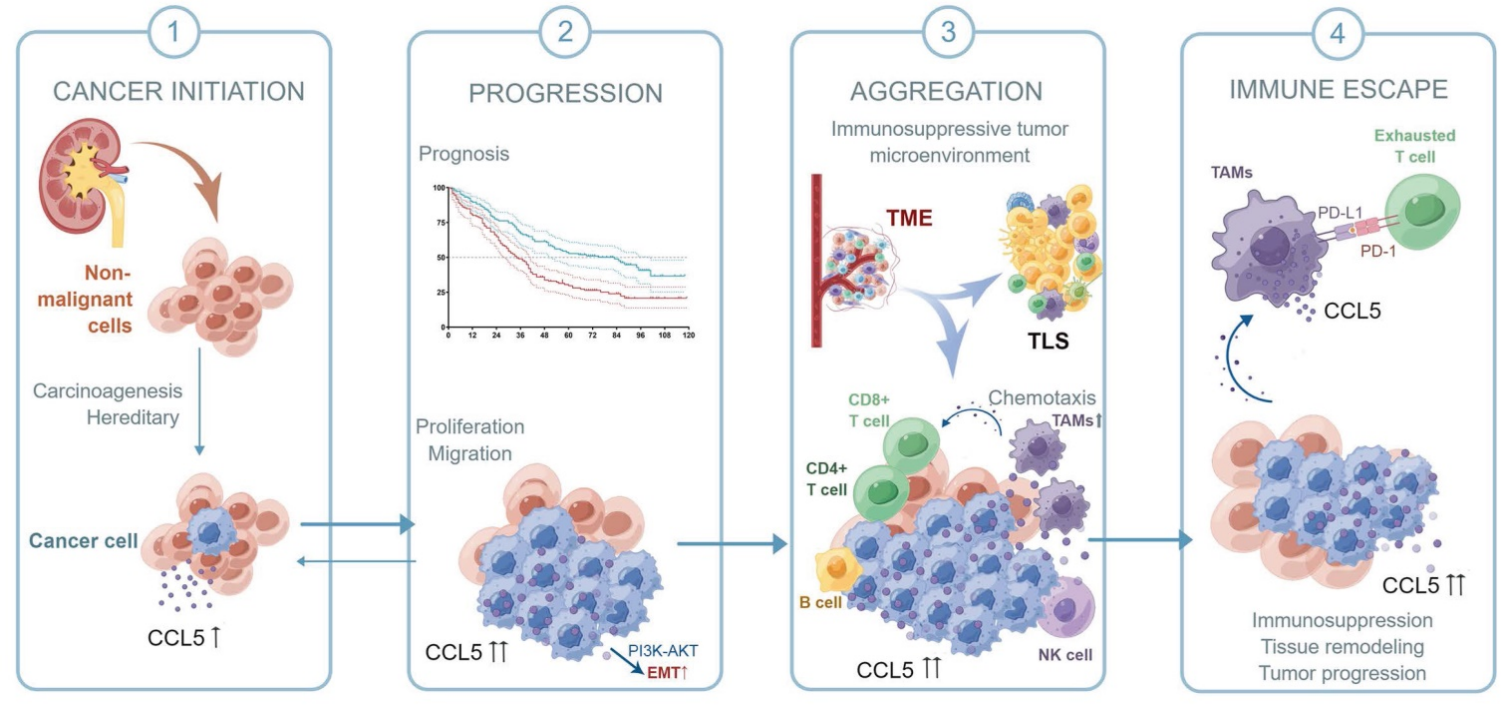
Schematic diagram of this study.

Collectively, in this study, we first confirmed the upregulation of CCL5 in ccRCC tissues and predicted poor outcomes for ccRCC patients. We speculated that by activating the PI3K/AKT pathway, CCL5 increased Snail1 expression, which led to the downregulation of the epithelial marker E-cadherin and upregulation of the mesenchymal markers Vimentin and Fibronectin, thus promoting the EMT, migration, and metastasis of ccRCC cells. Due to the specific role of CCL5 in the 12-chemokine signature guiding precision response to immune checkpoint blockade 50, we labeled TLS-related lymphocytes using a multi-label immunofluorescence assay for the identification and quantitative analysis of TILs in ccRCC TME. By labeling immune cells in different tumor localizations, we could distinguish the aggregated TILs as well as TLSs of the different subtypes and maturation levels. Samples with increased CCL5 expression exhibited a higher aggregation of TILs, but a prominently reduced proportion of immature/mature TLS and PD-L1^+^ CD68^+^ TAMs, revealing a typical suppressive TIME in ccRCC. Taken together, as shown in the schematic diagram of the hypothesis, intra-tumoral CCL5^+^ TAMs were able to predict a distinct pro-tumorigenic exhausted state of CD8^+^ T cells and promote the immunoevasive contexture of ccRCC (**Figure 8**). The infiltration level of pro-tumorigenic CCL5^+^ TAMs cells could serve as an independent predictor for evaluating the immunoevasive TME characteristic and clinical outcomes of ccRCC.

We recognize several limitations of this work. First, the retrospective nature of the three cohorts enrolled in this study necessitates future prospective validation. In addition, our research was not able to include in-depth experiments on the mechanism of the pro-tumorigenic role of CCL5 in ccRCC and CCL5^+^ TAMs in regulating an aberrant TME status, which might reflect the potential replicability issues in clustering patients with differential CCL5^+^ TAMs infiltration status.

## Conclusion

In conclusion, this study first revealed the predictive value of CCL5 on the progression and TIME of ccRCC. In clinical practice, the CCL5+ TAMs could be used to comprehensively evaluate the prognostic patterns as well as unique TME characteristics among individuals, allowing for the identification of different immunophenotypes and promotion of treatment efficiency for ccRCC. The comprehensive assessment of intra-tumoral CCL5^+^ TAMs could strengthen our understanding of the infiltration characteristics of TILs and help tailor precise immunotherapy and targeted combination strategies for individual ccRCC patients.

## Supplementary Material

Supplementary figures and tables 2-3.

Supplementary table 1.

Supplementary table 4.
