# Supplementary material for: Tumor-associated macrophage-derived chemokine CCL5 facilitates the progression and immunosuppressive tumor microenvironment of clear cell renal cell carcinoma
Source: Int J Biol Sci. 2022 Jul 18;18(13):4884–900. doi: 10.7150/ijbs.74647 (PMC9379407; doi:10.7150/ijbs.74647)
Supplement: Supplementary file 1 — Supplementary figures and tables 2-3. [file ijbsv18p4884s1.pdf]

Figure S1. Protein-protein interaction network of hub protein concerning CCL5 with minimum required interaction confidence set as 0.7 using String web tool.

Figure S2. Association between *CCL5* mRNA expression and clinicopathological factors in 533 patients with ccRCC from TCGA cohort using Chi-square test.

Figure S3. Prognostic implications of *CCL5* mRNA expression in patients among 33 cancers from TCGA cohort. (**A-B**) Prognostic value of CCL5 mRNA expression in predicting overall and progression-free survival was assessed using Cox regression analysis and visualized in forest plots. (**C-D**) Histogram showing the predictive value of CCL5 expression in predicting advanced clinical stages and aggressive pathological grade with *P* value converted into $-\log_{10}$(P-value) in the y-coordinate among 33 cancers from TCGA cohort.

Figure S4. Drug sensitivity analysis: the distribution of IC50 score for Axitinib, Pazopanib, Sorafenib and Sunitinib in ccRCC samples of differential CCL5 expression level.

Figure S5. KEGG functional enrichment analysis of CCL5 expression in ccRCC

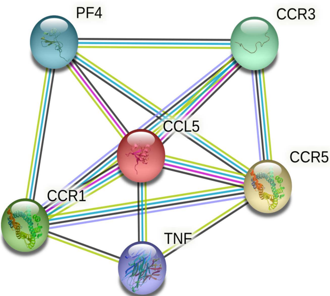
PF4
CCR3
CCL5
CCR5
CCR1
TNF

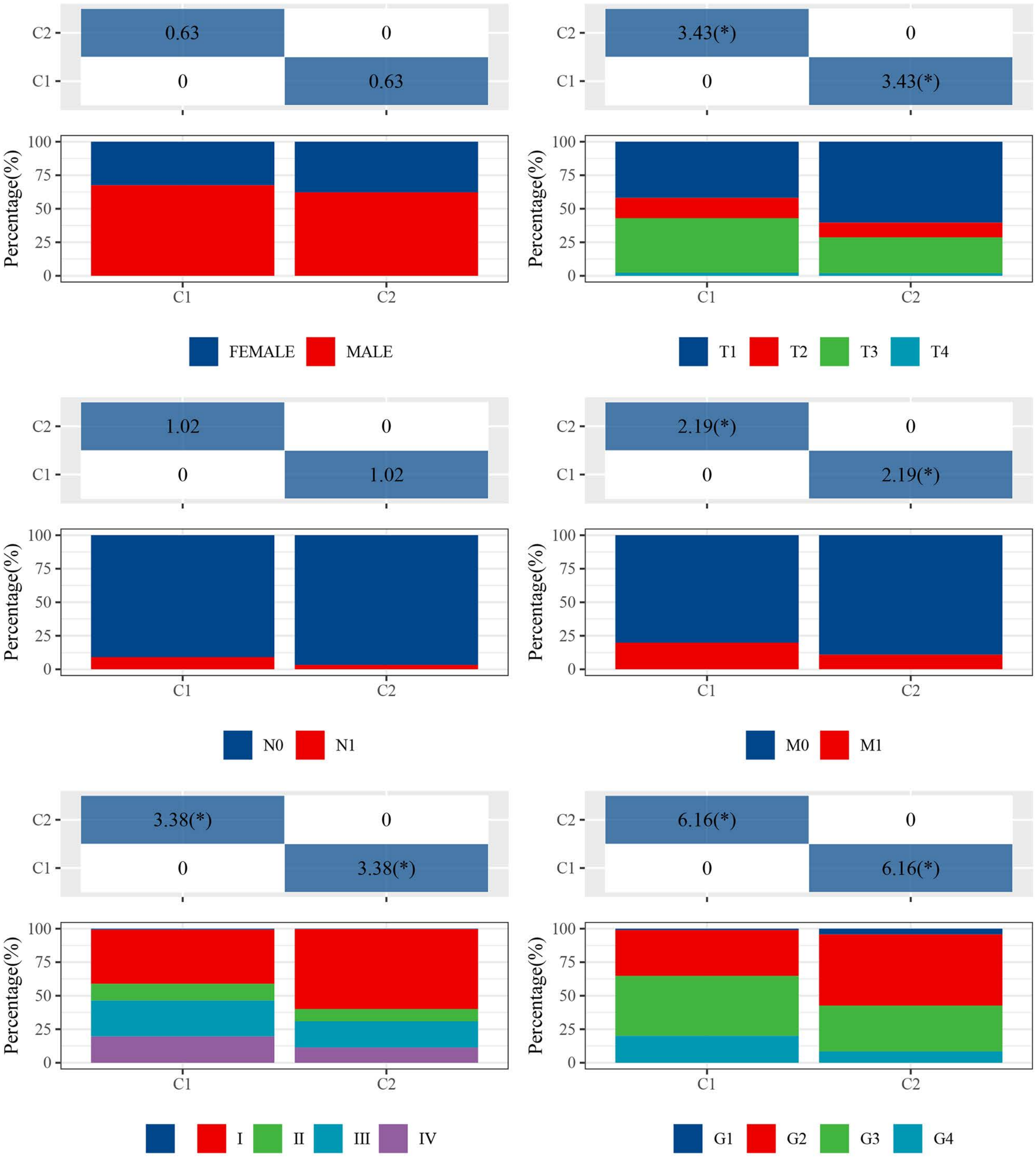

C2
C1
0.63
0
0
0.63
Percentage(%)
100
75
50
25
0
C1
C2
FEMALE
MALE
3.43(*)
0
0
3.43(*)
T1
T2
T3
T4
1.02
0
0
1.02
N0
N1
2.19(*)
0
0
2.19(*)
M0
M1
3.38(*)
0
0
3.38(*)
I
II
III
IV
6.16(*)
0
0
6.16(*)
G1
G2
G3
G4

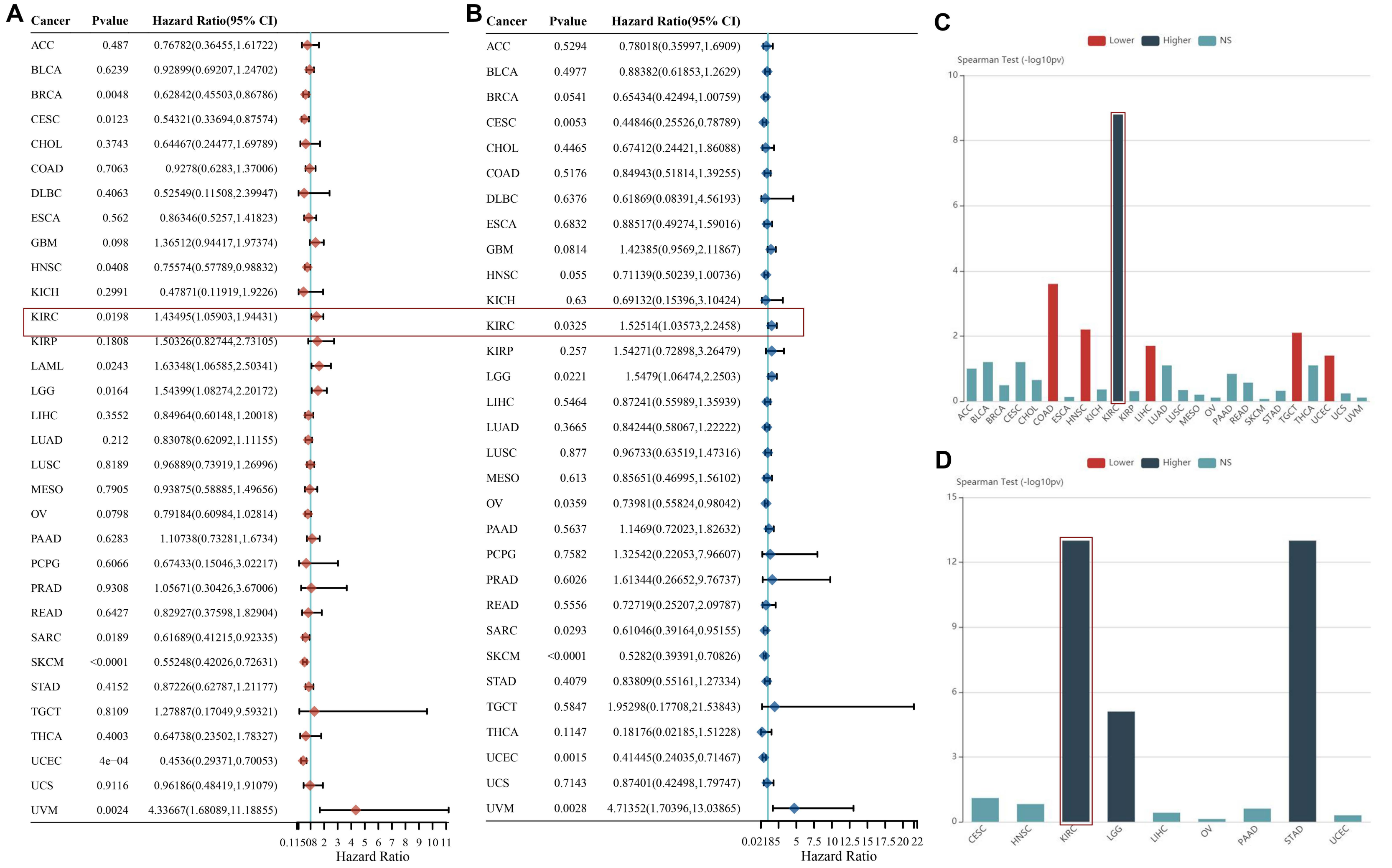
A
Cancer Pvalue Hazard Ratio(95% CI)
ACC 0.487 0.76782(0.36455,1.61722)
BLCA 0.6239 0.92899(0.69207,1.24702)
BRCA 0.0048 0.62842(0.45503,0.86786)
CESC 0.0123 0.54321(0.33694,0.87574)
CHOL 0.3743 0.64467(0.24477,1.69789)
COAD 0.7063 0.9278(0.6283,1.37006)
DLBC 0.4063 0.52549(0.11508,2.39947)
ESCA 0.562 0.86346(0.5257,1.41823)
GBM 0.098 1.36512(0.94417,1.97374)
HNSC 0.0408 0.75574(0.57789,0.98832)
KICH 0.2991 0.47871(0.11919,1.9226)
KIRC 0.0198 1.43495(1.05903,1.94431)
KIRP 0.1808 1.50326(0.82744,2.73105)
LAML 0.0243 1.63348(1.06585,2.50341)
LGG 0.0164 1.54399(1.08274,2.20172)
LIHC 0.3552 0.84964(0.60148,1.20018)
LUAD 0.212 0.83078(0.62092,1.11155)
LUSC 0.8189 0.96889(0.73919,1.26996)
MESO 0.7905 0.93875(0.58885,1.49656)
OV 0.0798 0.79184(0.60984,1.02814)
PAAD 0.6283 1.10738(0.73281,1.6734)
PCPG 0.6066 0.67433(0.15046,3.02217)
PRAD 0.9308 1.05671(0.30426,3.67006)
READ 0.6427 0.82927(0.37598,1.82904)
SARC 0.0189 0.61689(0.41215,0.92335)
SKCM <0.0001 0.55248(0.42026,0.72631)
STAD 0.4152 0.87226(0.62787,1.21177)
TGCT 0.8109 1.27887(0.17049,9.59321)
THCA 0.4003 0.64738(0.23502,1.78327)
UCEC 4e−04 0.4536(0.29371,0.70053)
UCS 0.9116 0.96186(0.48419,1.91079)
UVM 0.0024 4.33667(1.68089,11.18855)
0.11508 2 3 4 5 6 7 8 9 10 11
Hazard Ratio
B
Cancer Pvalue Hazard Ratio(95% CI)
ACC 0.5294 0.78018(0.35997,1.6909)
BLCA 0.4977 0.88382(0.61853,1.2629)
BRCA 0.0541 0.65434(0.42494,1.00759)
CESC 0.0053 0.44846(0.25526,0.78789)
CHOL 0.4465 0.67412(0.24421,1.86088)
COAD 0.5176 0.84943(0.51814,1.39255)
DLBC 0.6376 0.61869(0.08391,4.56193)
ESCA 0.6832 0.88517(0.49274,1.59016)
GBM 0.0814 1.42385(0.9569,2.11867)
HNSC 0.055 0.71139(0.50239,1.00736)
KICH 0.63 0.69132(0.15396,3.10424)
KIRC 0.0325 1.52514(1.03573,2.2458)
KIRP 0.257 1.54271(0.72898,3.26479)
LGG 0.0221 1.5479(1.06474,2.2503)
LIHC 0.5464 0.87241(0.55989,1.35939)
LUAD 0.3665 0.84244(0.58067,1.22222)
LUSC 0.877 0.96733(0.63519,1.47316)
MESO 0.613 0.85651(0.46995,1.56102)
OV 0.0359 0.73981(0.55824,0.98042)
PAAD 0.5637 1.1469(0.72023,1.82632)
PCPG 0.7582 1.32542(0.22053,7.96607)
PRAD 0.6026 1.61344(0.26652,9.76737)
READ 0.5556 0.72719(0.25207,2.09787)
SARC 0.0293 0.61046(0.39164,0.95155)
SKCM <0.0001 0.5282(0.39391,0.70826)
STAD 0.4079 0.83809(0.55161,1.27334)
TGCT 0.5847 1.95298(0.17708,21.53843)
THCA 0.1147 0.18176(0.02185,1.51228)
UCEC 0.0015 0.41445(0.24035,0.71467)
UCS 0.7143 0.87401(0.42498,1.79747)
UVM 0.0028 4.71352(1.70396,13.03865)
0.02185 5 7.5 10 12.5 15 17.5 20 22
Hazard Ratio
C
Lower Higher NS
Spearman Test (-log10pv)
10 8 6 4 2 0
ACC BLCA BRCA CESC CHOL COAD ESCA HNSC KICH KIRC KIRP LIHC LUAD LUSC MESO OV PAAD READ SKCM STAD TGCT THCA UCEC UCS UVM
D
Lower Higher NS
Spearman Test (-log10pv)
15 12 9 6 3 0
CESC HNSC KIRC LGG LIHC OV PAAD STAD UCEC

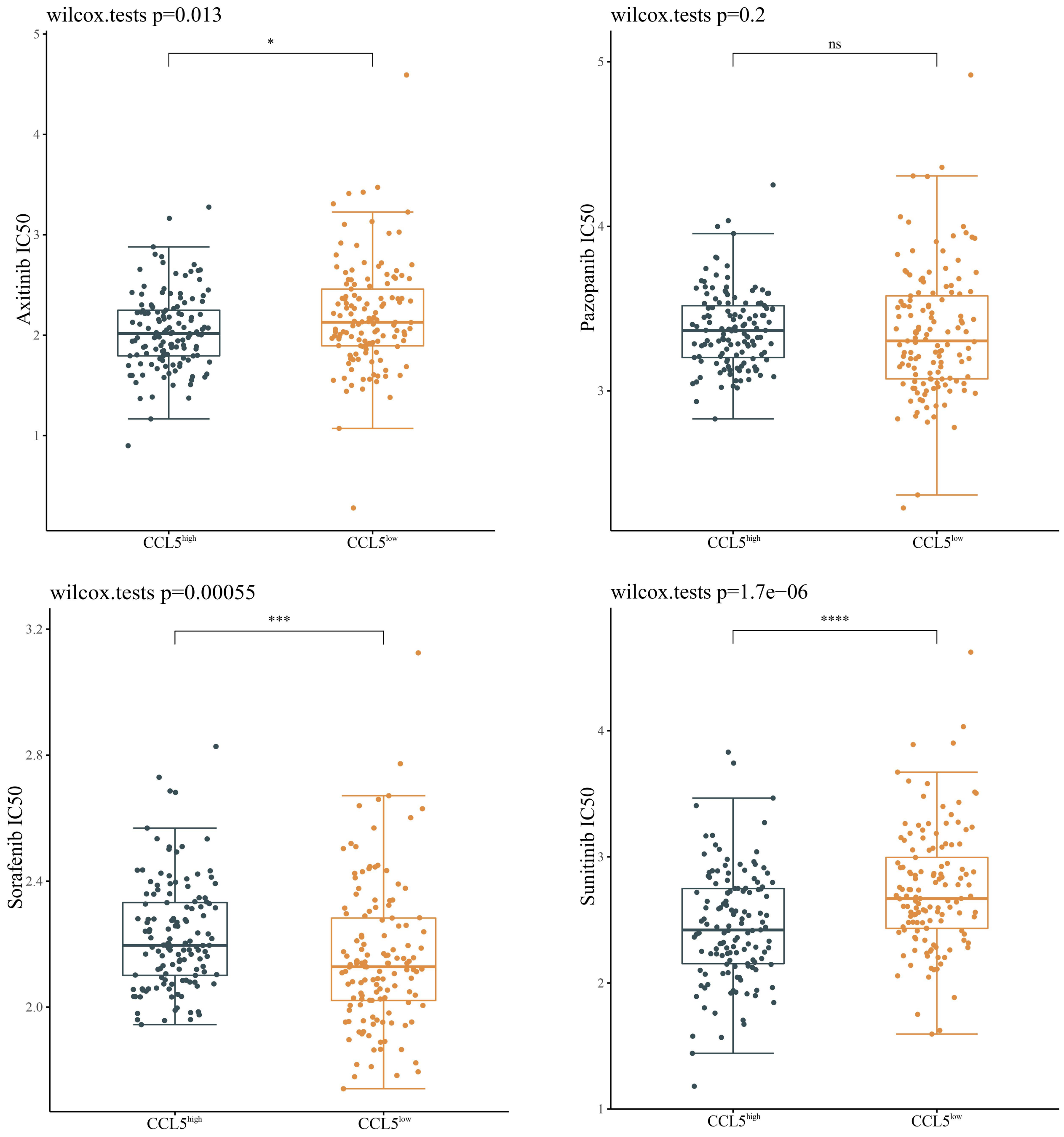

wilcox.tests p=0.013
*
Axitinib IC50
CCL5high
CCL5low
wilcox.tests p=0.2
ns
Pazopanib IC50
CCL5high
CCL5low
wilcox.tests p=0.00055
***
Sorafenib IC50
CCL5high
CCL5low
wilcox.tests p=1.7e−06
****
Sunitinib IC50
CCL5high
CCL5low

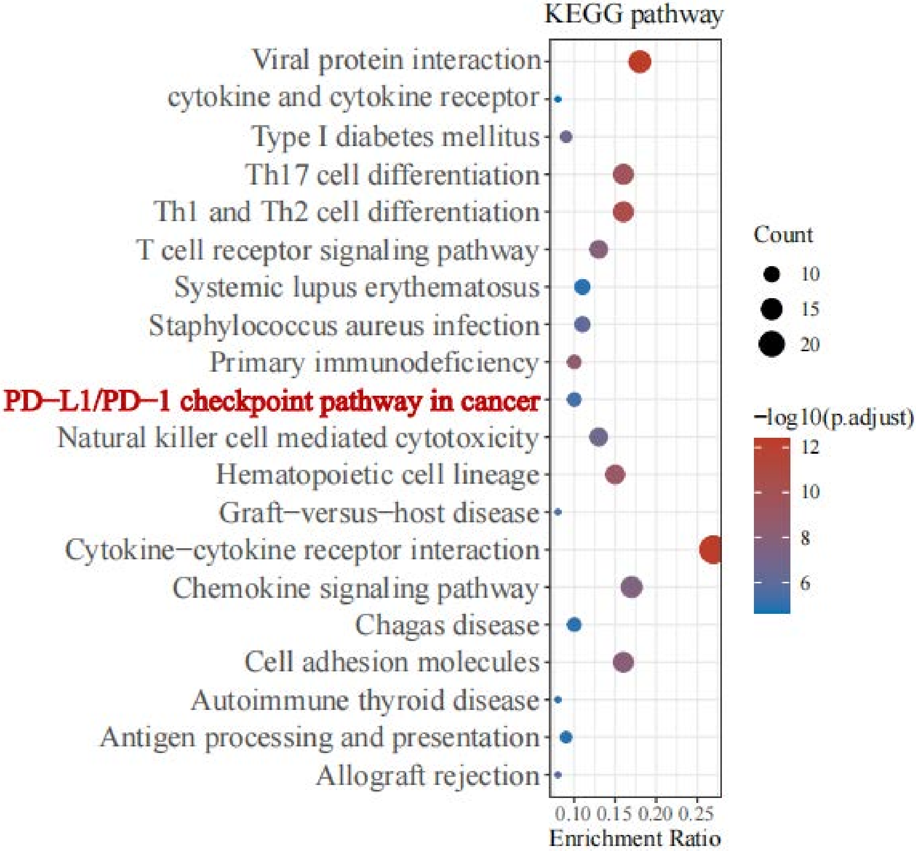
KEGG pathway
Viral protein interaction
cytokine and cytokine receptor
Type I diabetes mellitus
Th17 cell differentiation
Th1 and Th2 cell differentiation
T cell receptor signaling pathway
Systemic lupus erythematosus
Staphylococcus aureus infection
Primary immunodeficiency
PD−L1/PD−1 checkpoint pathway in cancer
Natural killer cell mediated cytotoxicity
Hematopoietic cell lineage
Graft−versus−host disease
Cytokine−cytokine receptor interaction
Chemokine signaling pathway
Chagas disease
Cell adhesion molecules
Autoimmune thyroid disease
Antigen processing and presentation
Allograft rejection
0.10 0.15 0.20 0.25
Enrichment Ratio
Count
10
15
20
−log10(p.adjust)
12
10
8
6

Supplementary Table 2. Antibodies used in immunohistochemistry, immunofluorescence and flow cytometry assays.

Markers	Antibody names	Provider	Host	Catalog No.
CCL5	Anti-Human CCL5 Antibody	CST	Rb	36467
CD68	Anti-Human CD68 Antibody	Abcam	Ms	ab955
CD45	APC-Cy7 Anti-Human CD45 Antibody	Biolegend	Ms	368516
CD45	PE-Cy7 Anti-Human CD45 Antibody	Biolegend	Ms	368532
CD14	FITC Anti-Human CD14 Antibody	BD	Ms	555397
CD3	PE-Cy7 anti-human CD3 Antibody	Biolegend	Ms	100220
CD4	Brilliant Violet® 421 Anti-Human CD4 Antibody	BD	Ms	562424
CD8	BB515 Anti-Human CD8 Antibody	BD	Ms	564526
CD19	PerCP/Cy5.5 anti-human CD19 Antibody	Biolegend	Ms	363016
CD11c	Brilliant Violet® 605 Anti-Human IL-2 Antibody	Biolegend	Ms	301636
CD80	PE-Cy7 Anti-Human CD80 Antibody	Biolegend	Ms	305218
CD86	Brilliant Violet® 650 Anti-Human CD86 Antibody	Biolegend	Ms	305428
CD68	Alexa Fluor® 647 Anti-Human CD68 Antibody	BD	Ms	562111
PRF-1	Alexa Fluor® 647 Anti-Human Perforin Antibody	BD	Ms	563576
CD69	CD69 (H1.2F3) Hamster mAb (FITC Conjugate)	CST	Rb	39443
TNF-α	Alexa Fluor® 647 anti-human TNF-α Antibody	Biolegend	Ms	502916
IFN-γ	IFN-γ (D3H2) XP® Rabbit mAb	CST	Rb	8455
PD-L1	PD-L1 (E1L3N®) XP® Rabbit mAb	CST	Rb	13684
Ki67	Ki-67 (D3B5) Rabbit mAb	CST	Rb	9129
GAPDH	GAPDH (D16H11) XP® Rabbit mAb	CST	Rb, Ms	5174
IgG	Goat Anti-Rabbit IgG conjugated with HRP	Abcam	Rb	ab205718
/	Human Exhausted T Cell Antibody Sampler Kit	CST	Rb, Ms	81490

Table S3. Univariate Cox regression analyses of PFS and OS in 290 enrolled ccRCC patients from FUSCC cohort.

	PFS		OS	
Covariates	HR (95%CI)	*P* value	HR (95%CI)	*P* value
Age at surgery	1.010 (0.996-1.023)	0.151	1.013 (0.999-1.027)	0.067
BMI (kg/m^2)	0.954 (0.690-1.318)	0.775	0.898 (0.636-1.268)	0.541
Sex (Ref. Male)	0.862 (0.623-1.192)	0.369	0.982 (0.700-1.379)	0.919
Laterality (Ref. left)	1.071 (0.794-1.444)	0.653	0.922 (0.667-1.274)	0.623
T stage (T1-T2)	1.451 (0.814-2.586)	0.207	**1.538 (1.006-2.341)**	**0.047**
N stage (Ref. N0)	**2.207 (1.384-3.520)**	**0.001**	**2.150 (1.325-3.490)**	**0.002**
M stage (Ref. M0)	**1.854 (1.123-3.058)**	**0.016**	**1.735 (1.004-2.998)**	**0.048**
ISUP grade (Ref. 1-2)	**2.030 (1.441-2.860)**	**<0.001**	**1.481 (1.017-2.157)**	**0.041**
AJCC stage(Ref. I-II)	**2.239 (1.095-4.581)**	**0.027**	**3.480 (1.641-7.382)**	**0.001**
CCL5 expression (Ref. Low)	**1.642 (1.209-2.230)**	**0.002**	**1.523 (1.092-2.124)**	**0.013**

Abbreviations: PFS: progression free survival; OS: overall survival; ccRCC: clear cell renal cell carcinoma; FUSCC, Fudan University Shanghai Cancer Center; HR: hazard ratio; CI: confidence interval; BMI: body mass index; ISUP: International Society of Urological Pathology

**P* value less than 0.05 was considered as statistically significance, and marked in bold.
